# Evaluating the bioactivity of a hydroxyapatite-incorporated polyetheretherketone biocomposite

**DOI:** 10.1186/s13018-019-1069-1

**Published:** 2019-01-25

**Authors:** Rui Ma, Dagang Guo

**Affiliations:** 1 Department of Orthopedics, The Second Affiliated Hospital of Xi’an Jiaotong University, Xi’an, 710004 Shaanxi China; 2 State Key Laboratory for Mechanical Behavior of Materials, School of Materials Science and Engineering, Xi’an Jiaotong University, Xi’an, 710049 China

**Keywords:** Polyetheretherketone, Hydroxyapatite, Composite, Bioactivity, Osseointegration

## Abstract

**Background:**

Polyetheretherketone (PEEK) exhibits stable chemical properties, excellent biocompatibility, and rational mechanical properties that are similar to those of human cortical bone, but the lack of bioactivity impedes its clinical application.

**Methods:**

In this study, hydroxyapatite (HA) was incorporated into PEEK to fabricate HA/PEEK biocomposite using a compounding and injection-molding technique. The tensile properties of the prepared HA/PEEK composites (HA content from 0 to 40 wt%) were tested to choose an optimal HA content. To evaluate the bioactivity of the composite, the cell attachment, proliferation, spreading and alkaline phosphatase (ALP) activity of MC3T3-E1 cells, and apatite formation after immersion in simulated body fluid (SBF), and osseointegration in a rabbit cranial defect model were investigated. The results were compared to those from ultra-high molecular weight polyethylene (UHMWPE) and pure PEEK.

**Results:**

By evaluating the tensile properties and elastic moduli of PEEK composite samples/PEEK composites with different HA contents, the 30 wt% HA/PEEK composite was chosen for use in the subsequent tests. The results of the cell tests demonstrated that PEEK composite samples/PEEK composite exhibited better cell attachment, proliferation, spreading, and higher ALP activity than those of UHMWPE and pure PEEK. Apatite islands formed on the HA/PEEK composite after immersion in SBF for 7 days and grew continuously with longer time periods. Animal tests indicated that bone contact and new bone formation around the HA/PEEK composite were more obvious than those around UHMWPE and pure PEEK.

**Conclusions:**

The HA/PEEK biocomposite created by a compounding and injection-molding technique exhibited enhanced osteogenesis and could be used as a candidate of orthopedic implants.

## Background

Polyaryletherketones (PAEKs) are a family of high-temperature thermoplastic polymers. The most common of these polymers that is used in the medical field is polyetheretherketone (PEEK), which is often applied as a material for an interbody fusion cage [1]. The chemical structure of the aromatic backbone molecular chains of PEEK confers exceptionally good chemical resistance and stability at high temperature or in the sterilization process [2]. Furthermore, PEEK is biocompatible, wear-resistant, and radiolucent [3], and the mechanical properties of PEEK are similar to those of human cortical bone, which prevents the stress-shielding effect that is frequently caused by metal implants [4]. However, PEEK is biologically inert and does not directly bind to bone [4, 5], which prevents its complete integration with the neighboring bone after implantation.

Hydroxyapatite (HA), which has a chemical composition of Ca_10_(PO_4_)_6_(OH)_2_, is the closest pure synthetic equivalent to human bone mineral [6]. Synthetic HA material is biocompatible and bioactive and clinically used as an important bone substitute [7]. HA possesses osteoconductive abilities as well as a remarkable ability to bind directly to bone [8, 9]. Therefore, HA is commonly coated onto a metal core or incorporated into polymers to prepare composites. The first HA-reinforced-polymer composite was micro-scale HA particle-reinforced high-density polyethylene (HDPE) developed by Bonfield and coworkers in the 1980s [10]. This composite containing 40 vol% HA was commercialized under the trade name HAPEX™ for use in non-load-bearing otologic and maxillofacial implants [11].

The strategy of adding HA fillers to make HA/PEEK composite to improve the bioactivity of PEEK has been proved to be feasible. HA has been incorporated into PEEK to prepare HA/PEEK composites using various techniques. Zhang et al. [12] manufactured HA/PEEK composites via a selective laser sintering technique and determined that the HA/PEEK composite supported cell growth of osteoblasts; in addition, composites with higher HA contents exhibited enhanced cell proliferation and osteogenic differentiation. Yu et al. [13] prepared HA/PEEK composite with 10, 20, 30, and 40 vol% HA by a mixing, compaction, and pressureless sintering process and evaluated the bioactivities of the HA/PEEK composites using the simulated body fluid (SBF) immersing test. The growth rate of the apatite increased with the HA volume fraction, suggesting that the bioactivity of the HA/PEEK composite increased with increasing HA content in the composite [13]. Ma et al. [14, 15] prepared HA/PEEK composite via an in situ synthetic process, and they demonstrated that the composite exhibited good biocompatibility and satisfactory bioactivity.

In the current study, micro-scale HA powders and PEEK powders were utilized to fabricate a HA/PEEK composite using a compounding and injection-molding technique. Theoretically speaking, higher HA content means higher bioactivity; however, the mechanical properties may be compromised with HA content increasing [16]. Therefore, we firstly evaluated the tensile properties of the 0–40 wt% HA/PEEK composites to choose an optimal HA content. Then, the bioactivity of the HA/PEEK composite was assessed and compared to that of ultra-high molecular weight polyethylene (UHMWPE) and pure PEEK by evaluating the osteoblast interactions, apatite-formation in vitro, and osseointegration in vivo.

## Methods

### Preparation of materials

**Fig. 1:**
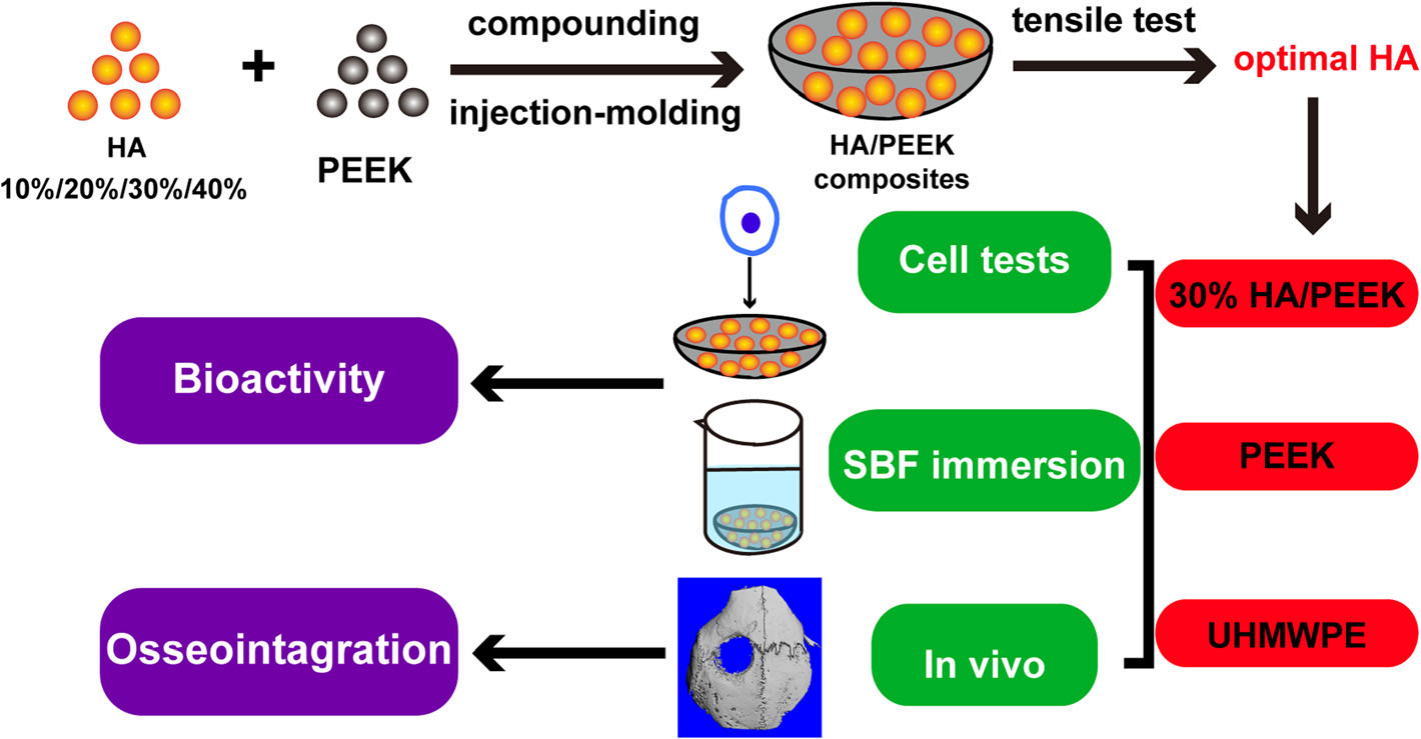
Schematic diagram of the preparation and biological evaluation of the HA/PEEK biocomposite

HA was prepared by a precipitation method using calcium hydroxide (Ca(OH)_2_, purity 99.0%; Sigma-Aldrich, USA) and phosphoric acid (H_3_PO_4_, purity 85.0%; Sigma-Aldrich, USA) [17]. PEEK powders with a mean particle size of 20 μm were used (Victrex, South Yorkshire, UK). The HA/PEEK composite samples with HA contents at 10, 20, 30, and 40 wt% were prepared via a compounding and injection-molding process (Fig. 1). The HA and PEEK powders were compounded in a high-speed ball mill (QM-3B, Nanjing T-Bota Scietech Instruments & Equipment Ltd., China) at a mixing speed of 500 rpm for 1 h, and then, the mixtures were dried at 150 °C for 24 h. The HA/PEEK composite samples were produced using an injection molding machine (BA-300/050CD, Battenfeld, Belgium) with an injecting temperature of 380 °C. In addition, the pure PEEK samples were prepared with PEEK powders via an injection and molding process. Ultra-high molecular weight polyethylene (UHMWPE; Roechling, Munich, Germany) with a molecular weight of two million served as the control. All the samples were cut into 2-mm-thick disks with a diameter of 15 mm.

### Tensile properties

The tensile tests were performed in accordance with ASTM D638 (ISO 527-1) with across head speed of 1.0 mm/min [18]. ASTM D638 is the test standard for tensile performance of plastic materials released by American Society for Testing Material (ASTM). The elastic modulus (E) and tensile strength (TS) were calculated. An average of five samples for each group was tested. All the tests were conducted at room temperature. By evaluating the tensile strengths and elastic moduli of the HA/PEEK composites with different HA contents, the 30 wt% HA/PEEK composite was chosen for use in the subsequent tests.

### Characterization of materials

The surface morphology and chemical composition of the 30 wt% HA/PEEK composite were characterized using a scanning electron microscope (SEM; S-4800, Hitachi, Japan) in back scattered electron mode and X-ray diffraction (XRD, S2000, Perkin-Elmer, USA). All the measurements were conducted at room temperature.

The hydrophilic properties of the material surfaces were determined by measuring the static water contact angles using the sessile drop method on a drop-shape analysis system (JC-2000D3, Shanghai Zhongcheng Digital Technology Co., China) at room temperature and ambient humidity. Five measurements were performed at different points on each sample.

### Cell culture

MC3T3-E1 cells were cultured in DMEM culture medium (Hyclone, Thermo, USA) supplemented with 10% fetal bovine serum (FBS; GibcoBRL, USA), 1% penicillin (100 U/ml; GibcoBRL, USA) and streptomycin sulfate (100 mg/ml; GibcoBRL, USA). The cells were cultured at 37 °C in a humidified atmosphere with 5% CO_2_. The culture medium was replaced every 2 days.

### Cell attachment and proliferation

A cell counting kit-8 (CCK-8) assay was used to study cell attachment and proliferation on surfaces of different material. In the cell attachment assay, the UHMWPE, PEEK, and HA/PEEK samples were previously placed in a 24-well plate (Costar, Corning, USA). The cultured MC3T3-E1 cells were digested with 0.25% trypsin (Sigma-Aldrich, USA), resuspended with culture medium and counted using a cell viability analyzer (Vi-cell XR, Beckman Counter Inc., USA), and seeded in each well in a density of 3 × 10^4^/cm^2^, with three empty wells containing 1 mL of DMEM set up as a blank control. The culture plates were incubated at 37 °C in a humidified atmosphere of 5% CO_2_. After incubation for 6, 12, and 24 h, all the samples were gently rinsed with phosphate-buffered saline (PBS) to remove the unattached cells, and 10 μL of a CCK-8 solution was added (Dojindo Molecular Technologies Inc., Japan), followed by incubation at 37 °C for 3 h. Then, the supernatant was transferred into a 96-well plate and read at 450 nm and 620 nm using a microplate reader (Synergy HT, Bio-tek, USA). The absorbance/optical density (OD) values of the test groups (OD_T_) were calculated as follows: OD_T_ = OD_450_ − OD_620_ − OD_B_ (mean ODs of the blank control). In the cell proliferation assay, the cell seeding density of the cells was 1 × 10^4^/cm^2^, and the time points were 1, 3, and 7 days.

### Cell spreading

The cell spreading was investigated by observing cells with SEM and detecting the filamentous actin of the cytoskeleton with a confocal laser scanning microscope (CLSM) after incubation for 24 h. To observe cells with the SEM, the MC3T3-E1 cells on the material surfaces were fixed with 2.5% glutaraldehyde for 15 min, dehydrated with gradient ethanol (30%, 50%, 70%, 80%, 90%, and 100%) for 10 min, treated with hexamethyldisilazane (HMDS; Sigma-Aldrich, USA) for 10 min, sputter-coated with gold, and observed with SEM (S-4800, Hitachi, Japan). To observe the cytoskeleton with CLSM, the cells were fixed with 4% paraformaldehyde for 15 min, permeabilized with 0.1% Triton X-100 for 10 min, and stained with rhodamine phalloidin (5 U/mL; Biotium, USA) for 30 min. The nuclei were counterstained with 4, 6-diamidino-2-phenylindole (DAPI, 0.1 μg/mL; Sigma-Aldrich, USA) for 10 min. The cytoskeletons and nuclei were visualized using a CLSM (Leica TCS SP2, Heidelberg, Germany).

### Alkaline phosphatase activity

After co-incubation with the specimens for 24 h, the culture medium was changed to the osteogenic inductive medium (DMEM supplemented with 10% FBS, 1% penicillin and streptomycin sulfate, 10 mM β-glycerophosphate sodium, 100 nM dexamethasone, and 50 μg/mL ascorbic acid). These media were renewed every 2 days. ALP staining was performed with an ALP staining kit (Shanghai Renbao Ltd., China) after 7 and 14 days, according to a previously published procedure [19]. The ALP activity was determined through a colorimetric assay based on p-nitrophenyl phosphate according to previously published procedures [20]. The total protein content was determined using the BCA protein assay kit (Pierce, Thermo, USA), and the ALP activity was normalized to the corresponding content of total protein.

### SBF immersion test

The ion concentrations of SBF is nearly equal to those of human blood plasma (Na^+^ 142.0 mM, K^+^ 5.0 mM, Mg^2+^ 1.5 mM, Ca^2+^ 2.5 mM, Cl^−^ 147.8 mM, HCO^3−^ 4.2 mM, HPO_4_^2−^ 1.0 mM, SO_4_^2−^ 0.5 mM, pH 7.40 at 36.5 °C) [21]. The SBF solution was prepared according to the Kokubo’s protocol [21]. The PEEK and 30 wt% HA/PEEK samples were immersed in 40 mL of SBF at 37 °C in a humidified atmosphere. After 7, 14, 21, and 28 days, the samples were rinsed three times with distilled water and dried at 37 °C overnight. Then, the samples were observed and analyzed with SEM and energy dispersive spectrometry (EDS; X-Max, Horiba, Japan). Furthermore, the concentrations of Ca and P ions in the soaked SBF were measured by inductively coupled plasma atomic emission spectroscopy (ICP-AES; Varian, USA).

### In vivo study

Thirty mature female New Zealand white rabbits weighting 2.4 ± 0.2 kg were divided into three groups as follows: group A (UHMWPE), group B (PEEK), and group C (HA/PEEK). The operative procedures and animal care were performed according to the ethical principles of the Second Affiliated Hospital of Xi’an Jiaotong University on animal experimentation (approval number: 2016105). The anesthesia was induced by intramuscular injection of 2% xylazine (12 mg/kg) and ketamine (80 mg/kg). A longitudinal incision was made down to the periosteum from the nasal bone to the occipital protuberance at the parietal bone, and the periosteum was undermined and lifted off the parietal skull. The skull defect was drilled in the middle of the parietal bone with an outer diameter of 8.0 mm using a trephine (Med-X Research Institute of Shanghai Jiaotong University, China). Finally, the samples with a diameter of 8 mm were implanted into the defect. At 3 weeks, 2 weeks, and 1 week prior to being sacrificed, the rabbits were intramuscularly injected with alizarin red solution (30 mg/kg), calcein solution (20 mg/kg), and alizarin red solution (30 mg/kg), respectively. After feeding for 8 weeks, all the rabbits were sacrificed, and the parietal bones were retrieved, soaked into 75% ethanol for 1 week, and dehydrated with gradient ethanol (80%, 95%, and 100%).

The samples were embedded in methylmethacrylate and cut into 150 μm sections with a microtome (SP 1600, Leica, Germany). Then, the sections were then adhered to organic glass slides; compressed for 24 h; polished to 50 μm using P300, P800, and P1200 abrasive paper; and then burnished with flannelette and abradum to 20–30 μm. At least three sections of each implant were stained with picric acid/fuchsine. A light microscope (Leica Microsystems AG, Germany) was used for histological evaluation. The fluorescence of the new bone formation labeled with alizarin red and calcein was visualized using a CLSM (TCS SP2, Leica Microsystems, Germany).

### Statistical analysis

All the data are presented as the means ± standard deviations. All the in vitro experiments were conducted in triplicate and repeated three times. Statistical significances between different groups were analyzed using a one-way analysis of variance (ANOVA) test, and multiple comparisons were performed using the least significant difference (LSD) test to determine any significant differences between each pair of groups. *p* < 0.05 was considered statistically significant, and *p* < 0.01 was considered highly statistically significant.

## Results

**Fig. 2:**
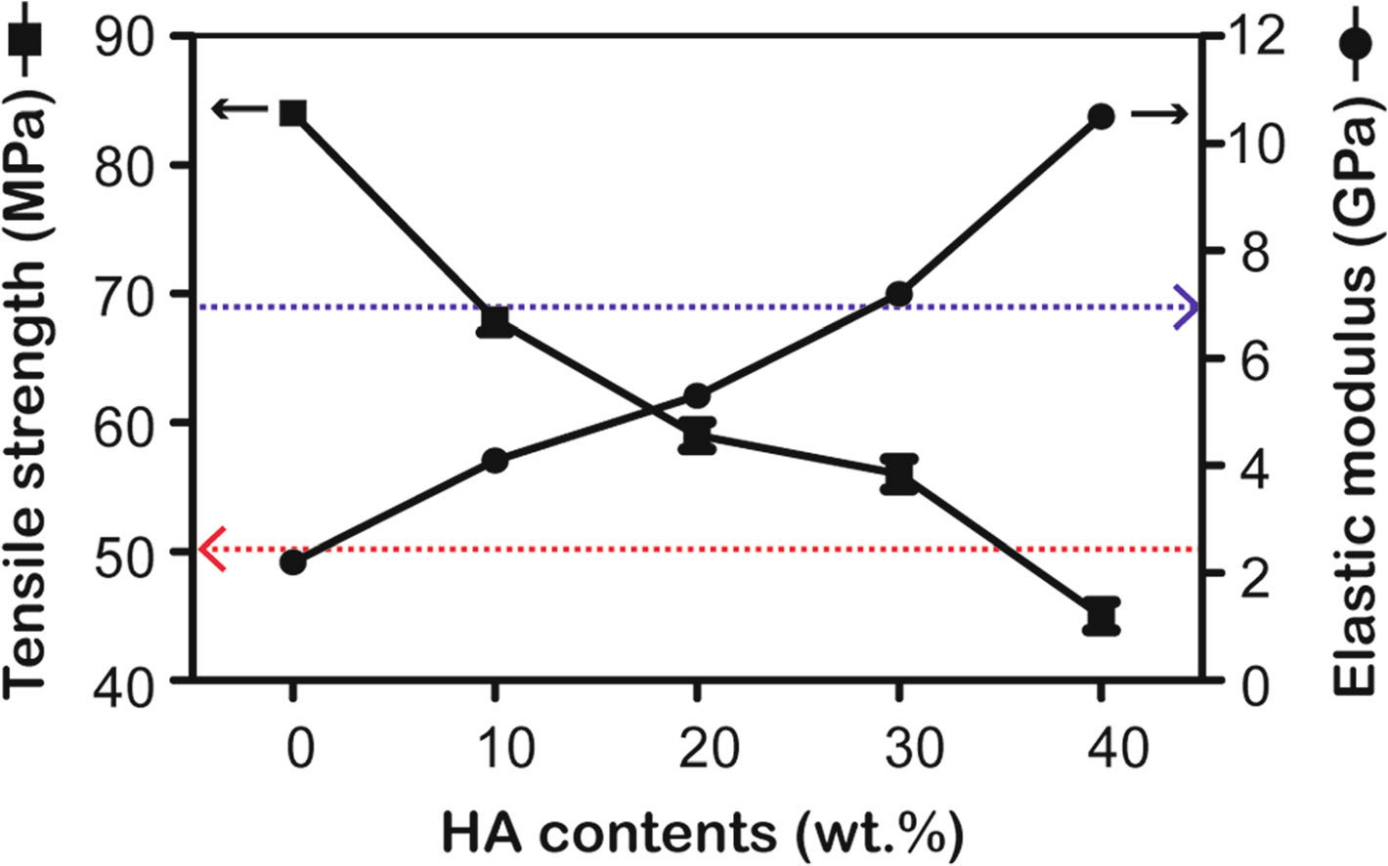
Effect of the HA content on the tensile strength and elastic modulus of the HA/PEEK composites. The blue dotted line represents the elastic modulus of the lower range of cortical bone (7 GPa). The red dotted line represents the tensile strength of the lower range of cortical bone (50 MPa)

Figure 2 shows the tensile property results for the HA/PEEK composites. An increase in the amount of HA (from 0 to 40%) resulted in an increase in the elastic modulus and a decrease in the tensile strength. In comparison with PEEK, the elastic moduli of the HA/PEEK composites with HA contents of 10%, 20%, 30%, and 40% increased by approximately 86%, 141%, 241%, and 468%, respectively. The elastic moduli of the HA/PEEK composites (30% and 40%) were higher than that of the lower range of cortical bone (7 GPa) [22]. When the HA contents were equal to or less than 30%, the tensile strengths of the HA/PEEK composites were higher than that of the lower range of cortical bone (50 MPa) [22]. However, the tensile strength of the 40% HA/PEEK composite (45 ± 2.5 MPa) was lower than 50 MPa, which was not compatible with cortical bone. By evaluating the tensile properties and elastic moduli of the HA/PEEK composites with different HA contents, a HA content of 30 wt% was chosen as an optimal HA content.

**Fig. 3:**
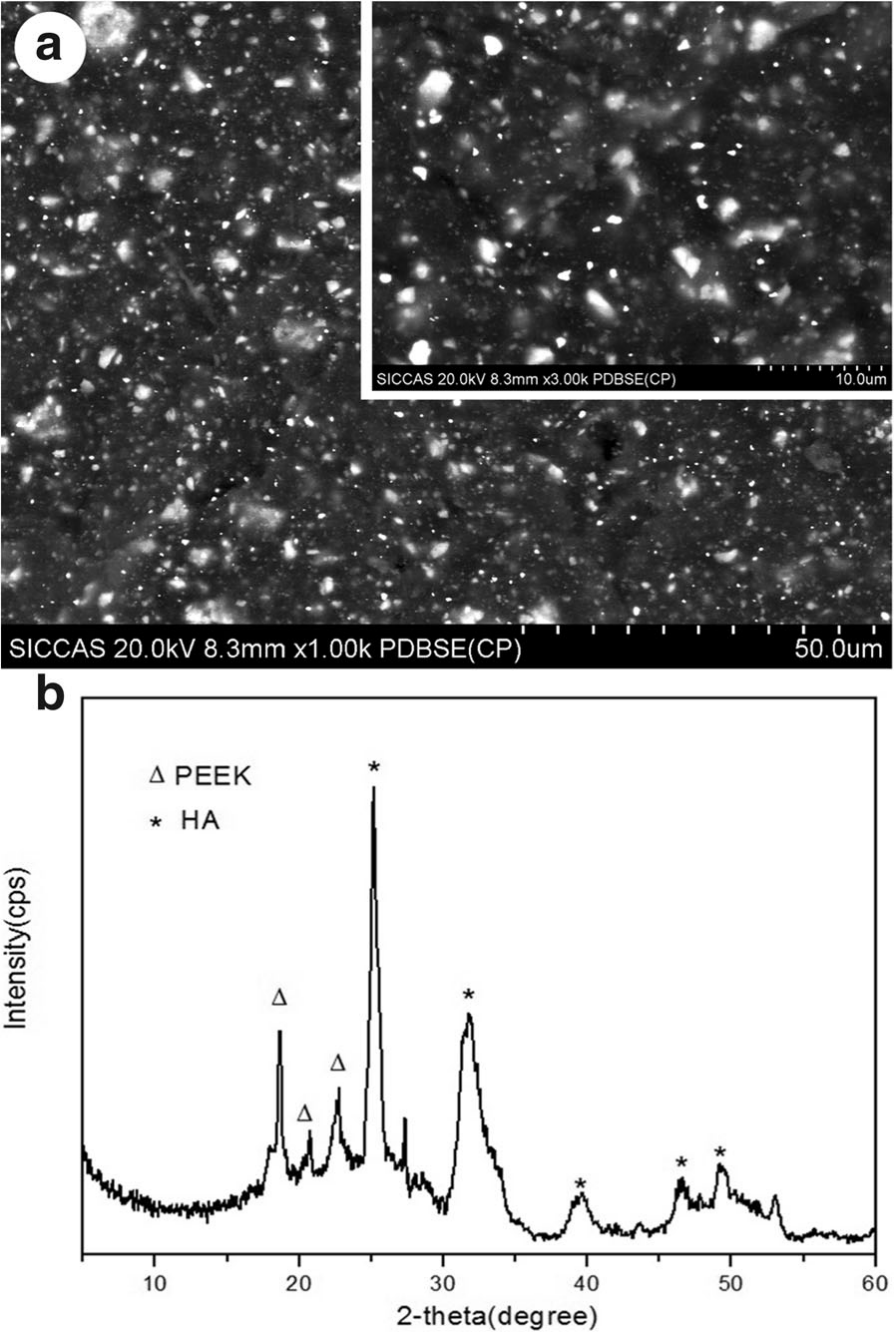
SEM images (**a**) and XRD pattern (**b**) of the HA/PEEK composite

Figure 3 shows the surface morphology (Fig. 3a) and phase composition (Fig. 3b) of the HA/PEEK surface observed by SEM and XRD. Numerous HA particles (i.e., white spots) were uniformly distributed on the material surface. The diffraction peaks of the composite at approximately 2θ = 18.5°, 21°, and 22.5° corresponded to the characteristic peaks of PEEK [23], and the peaks at approximately 2θ = 25.5°, 32°, 40°, 46.5°, and 49.5° were the characteristic peaks of HA [24]. The results indicated that the HA/PEEK composite was composed of HA and PEEK.

**Fig. 4:**
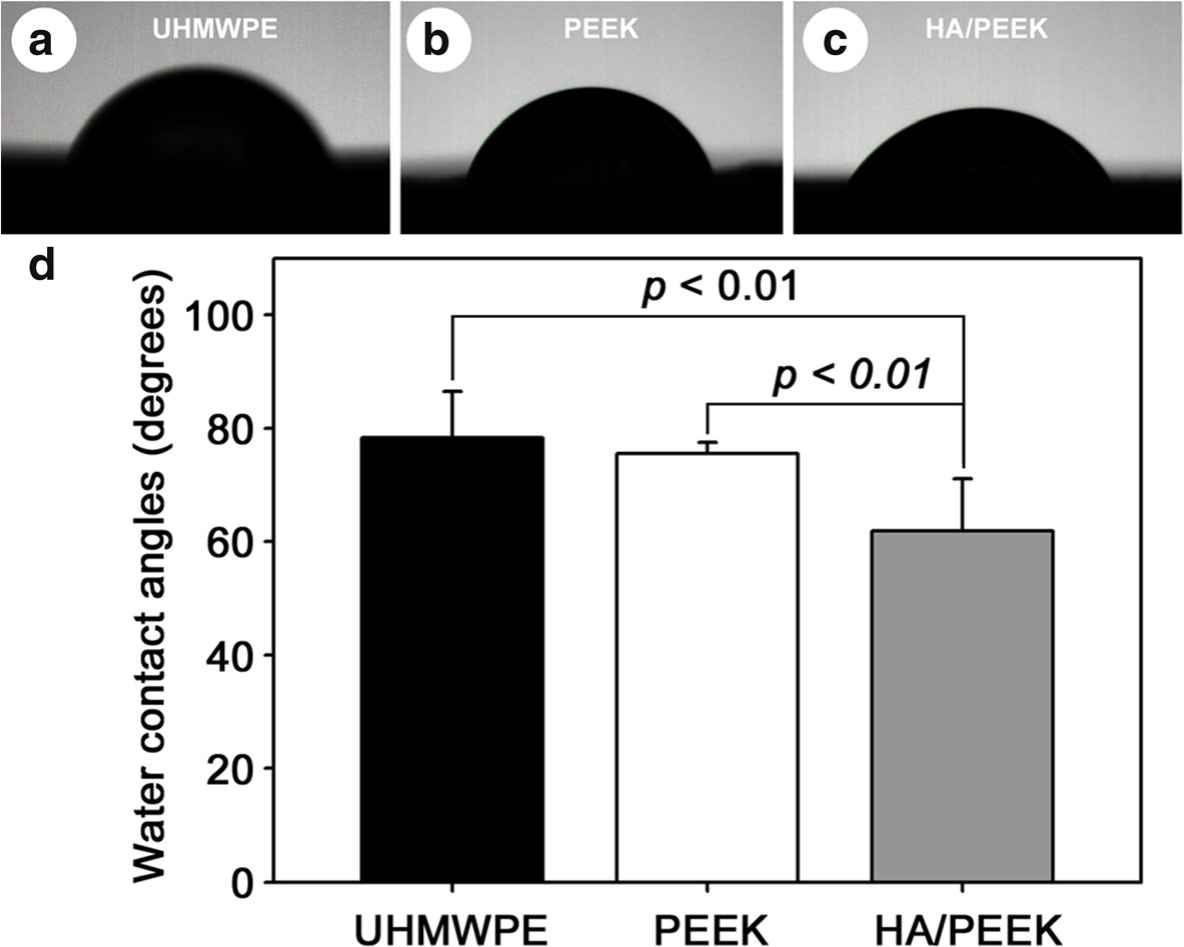
Measurement of water contact angles: **a** UHMWPE, **b** PEEK, **c** HA/PEEK, and **d** statistical comparison

The hydrophilic properties of the material surfaces were evaluated by measuring the water contact angles. The water contact angles of UHMWPE, pure PEEK, and HA/PEEK were 78.3 ± 8.1°, 75.6 ± 1.9°, and 61.9 ± 9.2°, respectively (Fig. 4). The water contact angle of the HA/PEEK composite was significantly lower than those of UHMWPE and pure PEEK (*p* = 0.006; Fig. 4d), indicating that the HA/PEEK composite was more hydrophilic than UHMWPE and pure PEEK.

**Fig. 5:**
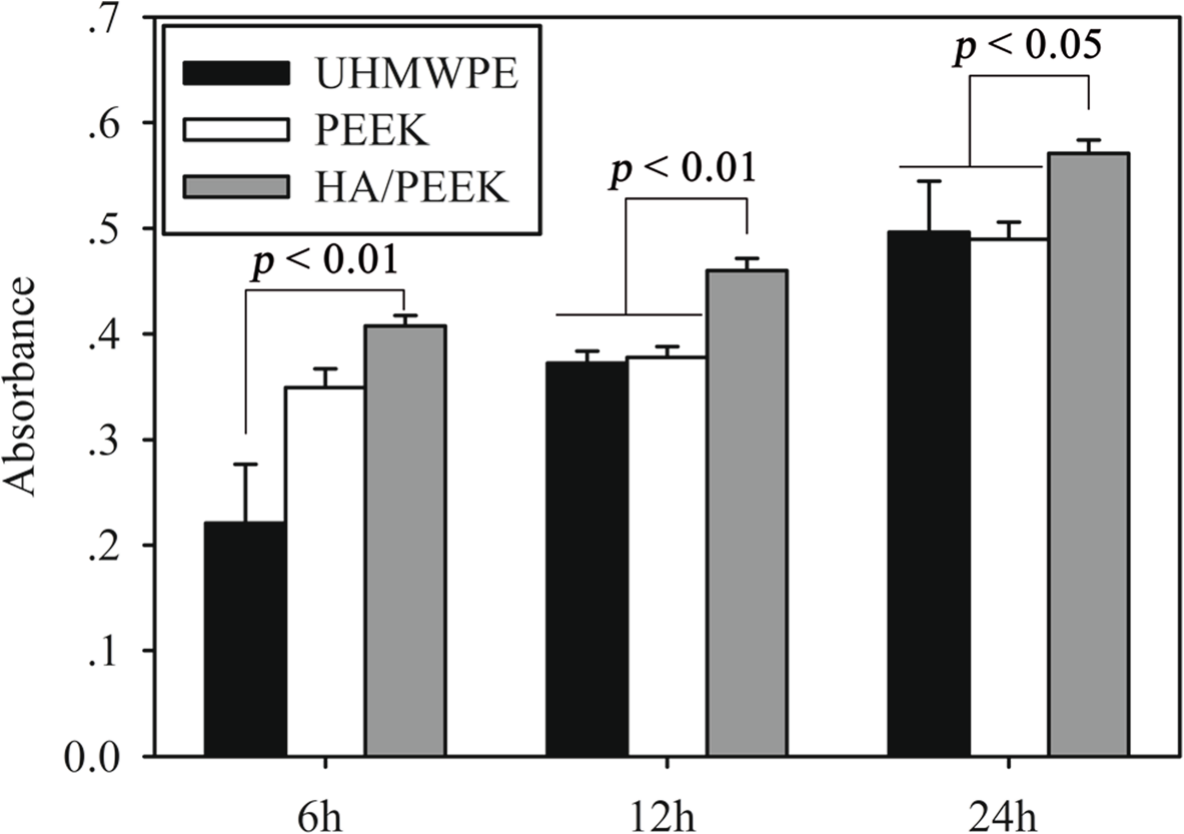
Cell attachment on different material surfaces after 6, 12, and 24 h

Figure 5 shows the cell attachment results from the CCK-8 assay. The number of adhered cells on the HA/PEEK composite was significantly higher than that on UHMWPE at 6 h (*p* = 0.001), 12 h (*p* = 0.000), and 24 h (*p* = 0.024). Furthermore, more cells were adhered on HA/PEEK than on PEEK at 12 h (*p* = 0.000) and 24 h (*p* = 0.017).

**Fig. 6:**
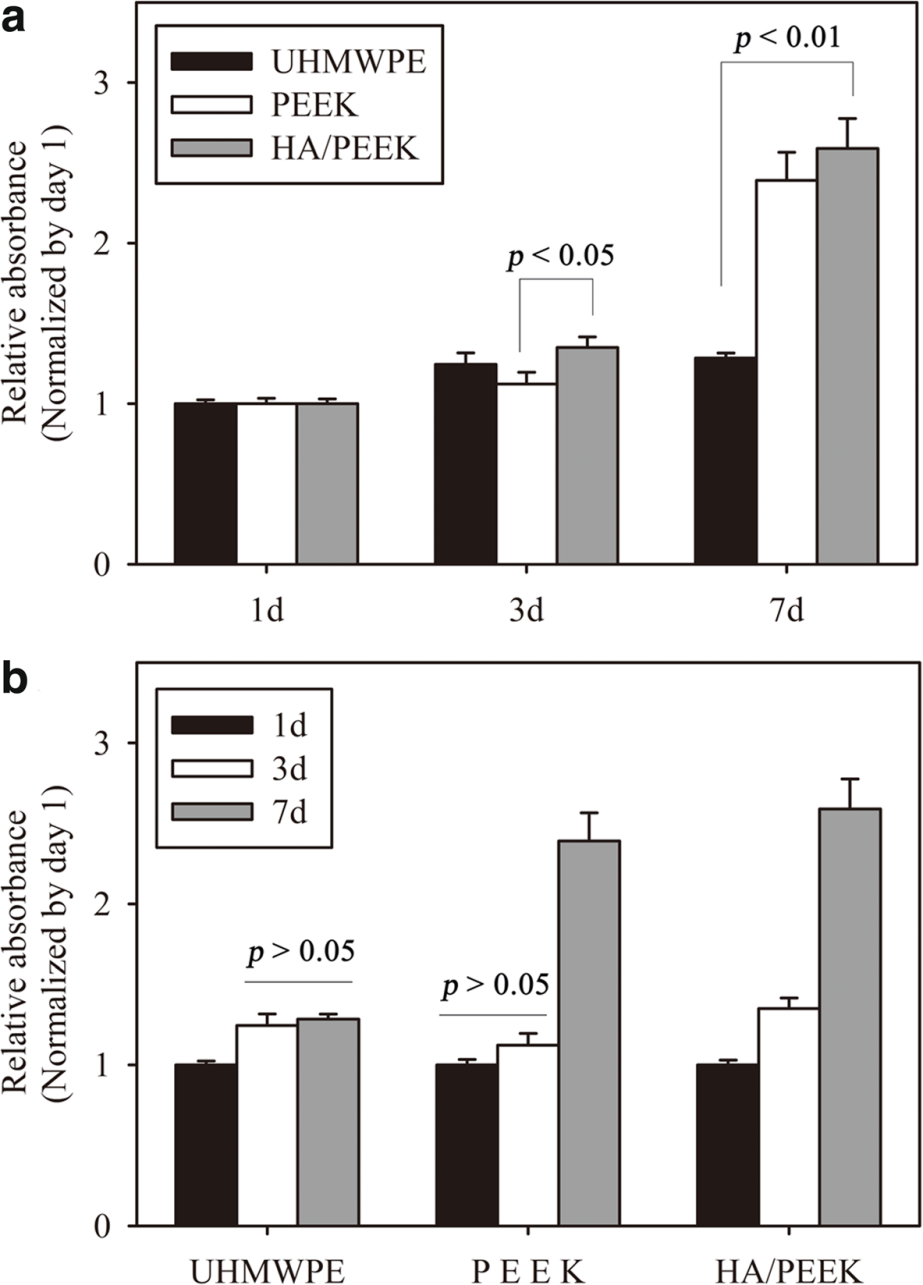
Cell proliferation on different material surfaces after 1, 3, and 7 days: **a** relative absorbance of MC3T3-E1 cells and **b** proliferative tendency of MC3T3-E1 cells from day 1 to day 7. The absorbance values at day 3 and day 7 were normalized to those at day 1

The results of the cell proliferation cultured on the material surfaces are shown in Fig. 6. The cells cultured on the HA/PEEK composite exhibited a higher relative absorbance than UHMWPE at day 7 (*p* = 0.003) and PEEK at day 3 (*p* = 0.032). The MC3T3-E1 cells on the HA/PEEK composite exhibited an increasing tendency from day 1 to day 7 (Fig. 6b). However, the cells on the UHMWPE from day 3 to day 7 and the cells on the PEEK from day 1 to day 3 exhibited no apparent increasing tendency (*p* = 0.081).

**Fig. 7:**
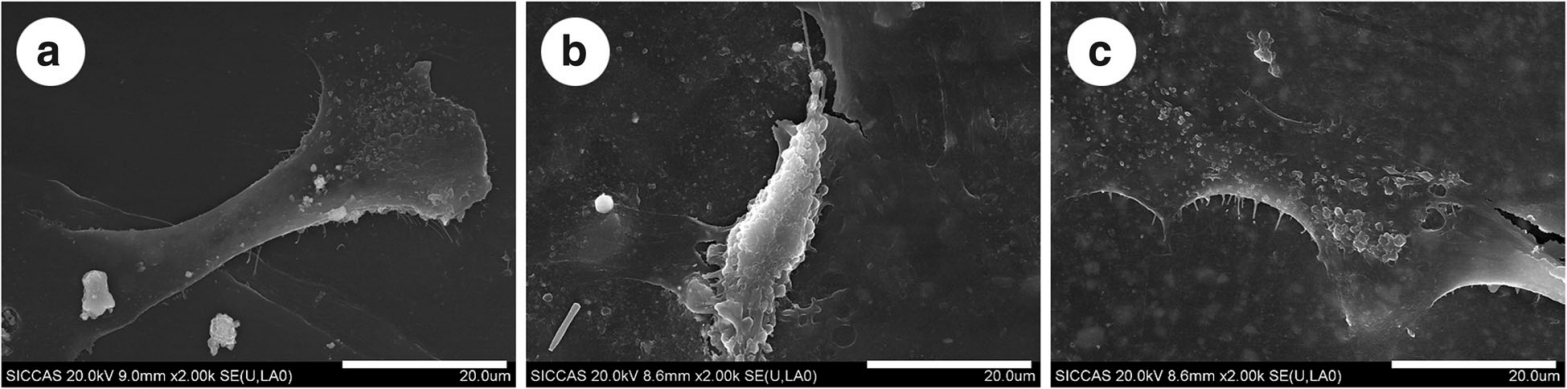
SEM images of MC3T3-E1 cells cultured on different material surfaces after 24 h: **a** UHMWPE, **b** PEEK, and **c** HA/PEEK. The scale bar represents 20 μm

Figure 7 shows the SEM images of the single cells cultured on different material surfaces. The cells on the surface of HA/PEEK were polygonal with numerous pseudopods anchored at the material surface after 24 h (Fig. 7c), while the cells on the UHMWPE (Fig. 7a) and pure PEEK (Fig. 7b) were rod-like with few pseudopods, indicating that the cell spreading on UHMWPE and pure PEEK was less efficient than that on the HA/PEEK composite.

**Fig. 8:**
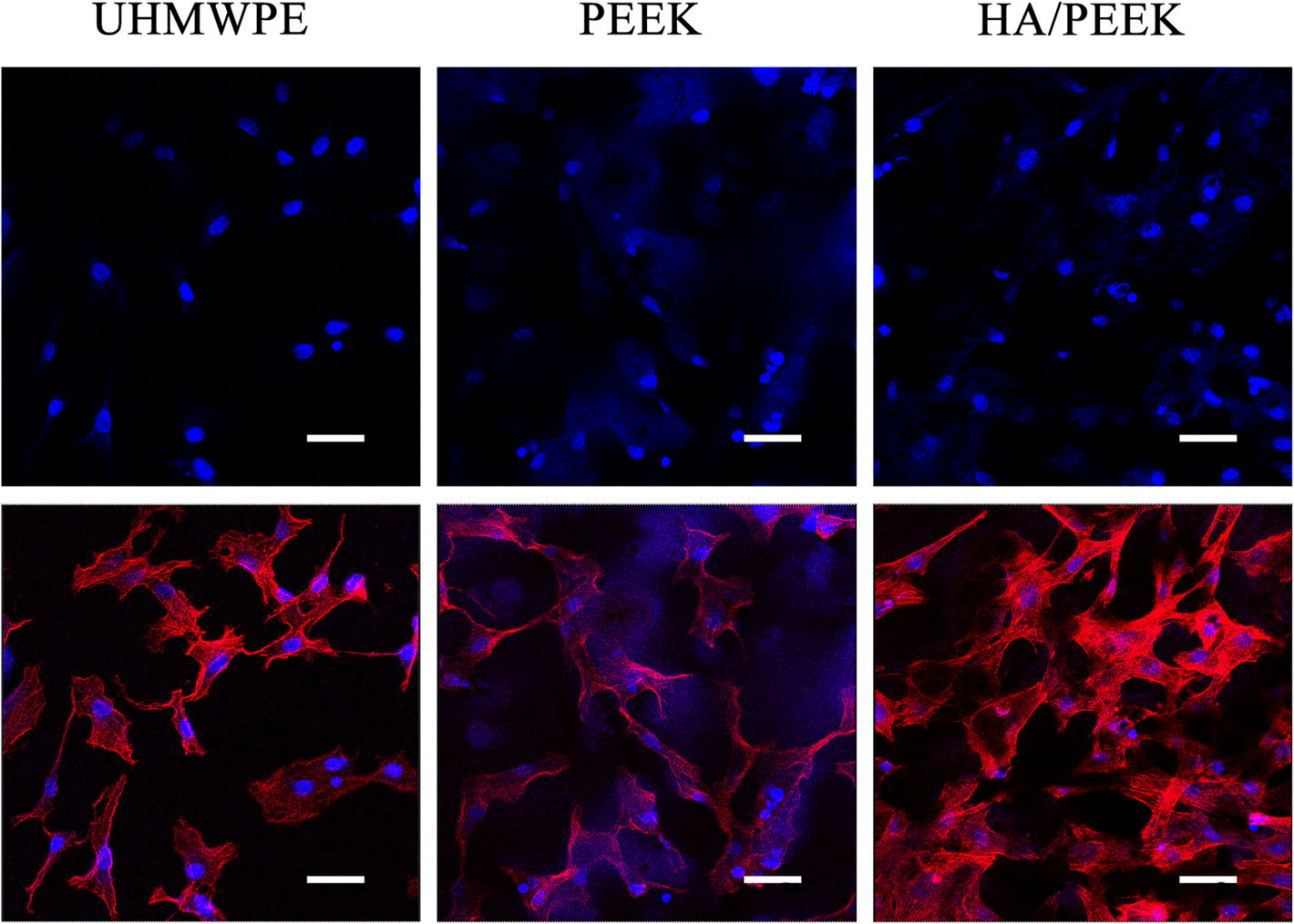
CLSM images showing the cytoskeletal morphology. The upper row shows nuclei staining by DAPI (blue), and the lower row shows cytoskeleton stained by rhodamine-phalloidin (red) and nuclei counterstained with DAPI (blue). The scale bar represents 50 μm

The CLSM images showing the cytoskeletal morphology are presented in Fig. 8. The cells on the HA/PEEK composite were confluent and clustering, but the cells on the UHMWPE and PEEK were dispersive. More obvious actin filaments that linked adjacent cells were observed on HA/PEEK. Additionally, the cell nuclei on the HA/PEEK composite were denser than those on the UHMWPE and PEEK surfaces.

**Fig. 9:**
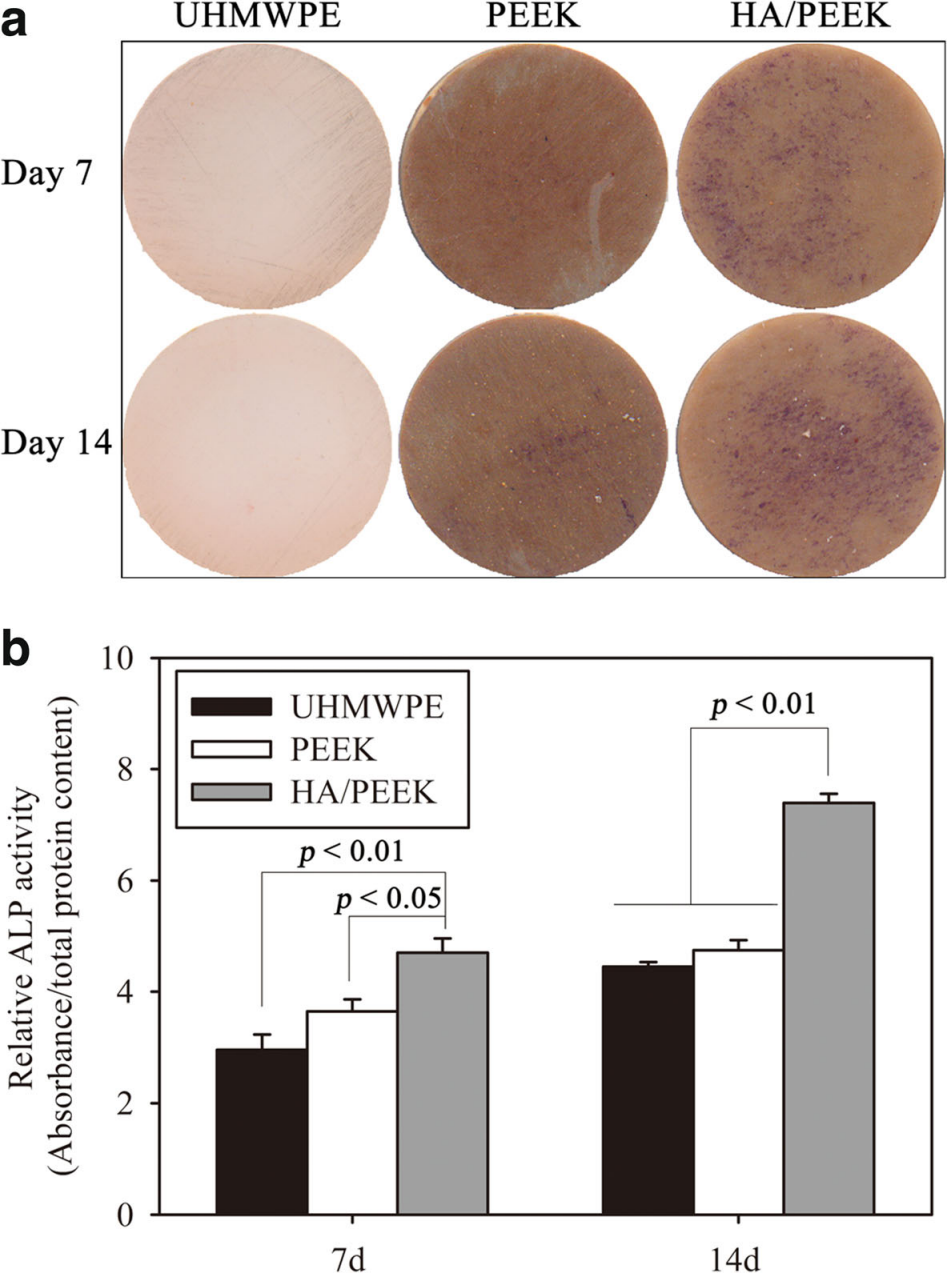
ALP staining and ALP activity of different material surfaces after 7 and 14 days: **a** ALP staining and **b** relative ALP activity of MC3T3-E1 cells

The qualitative and quantitative results of the ALP activity are shown in Fig. 9. In Fig. 9a, plenty of ALP staining points (purple color) were observed on the HA/PEEK composite, and these points were denser than those on the UHMWPE and PEEK surfaces at each time point. The relative ALP activity of MC3T3-E1 cells on the HA/PEEK composite was significantly higher than those on UHMWPE (*p* = 0.004) and PEEK (*p* = 0.035 at day 7, *p* = 0.005 at day 14, Fig. 9b) at each time point, which was in agreement with the qualitative staining.

**Fig. 10:**
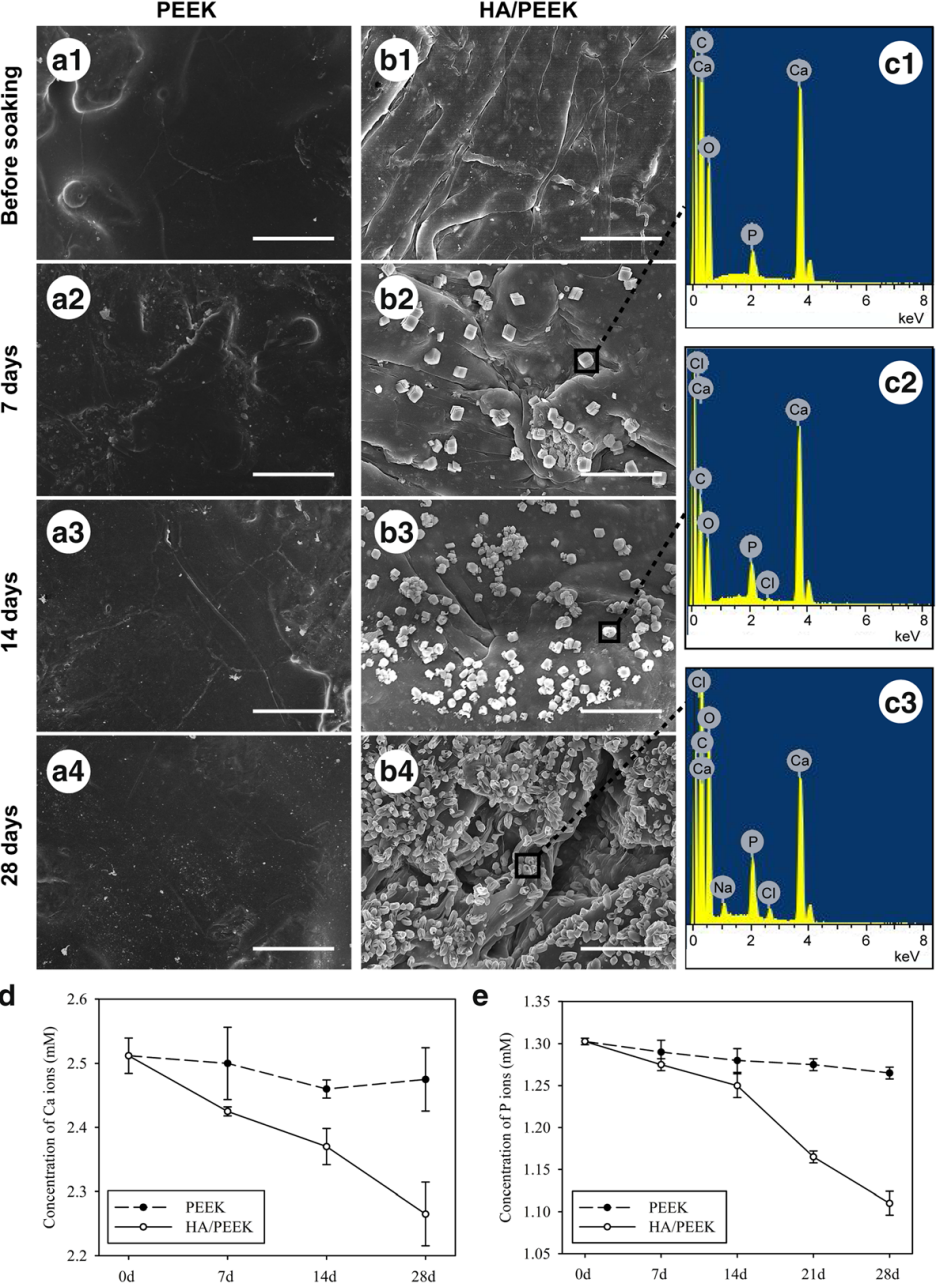
SBF immersion test showing the apatite-formation ability of the PEEK and HA/PEEK composite: **a1**–**a4** SEM images of PEEK, **b1**–**b4** SEM images of HA/PEEK, **c1**–**c3** EDS spectra of the apatite formed on the HA/PEEK surfaces, **d** concentration of Ca ions in the soaked SBF, and **e** concentration of P ions in the soaked SBF. The scale bar represents 10 μm

Figure 10 shows the SEM images and EDS spectra of the PEEK and HA/PEEK material surfaces before and after immersion in SBF for 7, 14, and 28 days. Even after 28 days of immersion in SBF, no newly formed substance was observed on the PEEK surface (Fig. 10a1–a4). However, a few square apatite islands formed on the surface of the HA/PEEK composite at day 7 (Fig. 10b2). When the immersion time was extended to 14 days, the apatite islands grew in number and size (Fig. 10b3). After 28 days, a mass of apatite islands formed and covered most areas of the HA/PEEK surfaces (Fig. 10b4). The newly formed islands on the HA/PEEK composite at 7, 14, and 28 days exhibited Ca and P peaks in the EDS spectra (Fig. 10c1–c3). The Ca and P concentrations in the PEEK-soaked solution exhibited a stable tendency. The concentration of Ca ions in the HA/PEEK-soaked SBF decreased incrementally from days 7 to 28 (Fig. 10d). The concentration of P ions in the HA/PEEK-soaked SBF decreased slowly from day 0 to day 14, and then decreased dramatically from day 14 to day 28 (Fig. 10e).

**Fig. 11:**
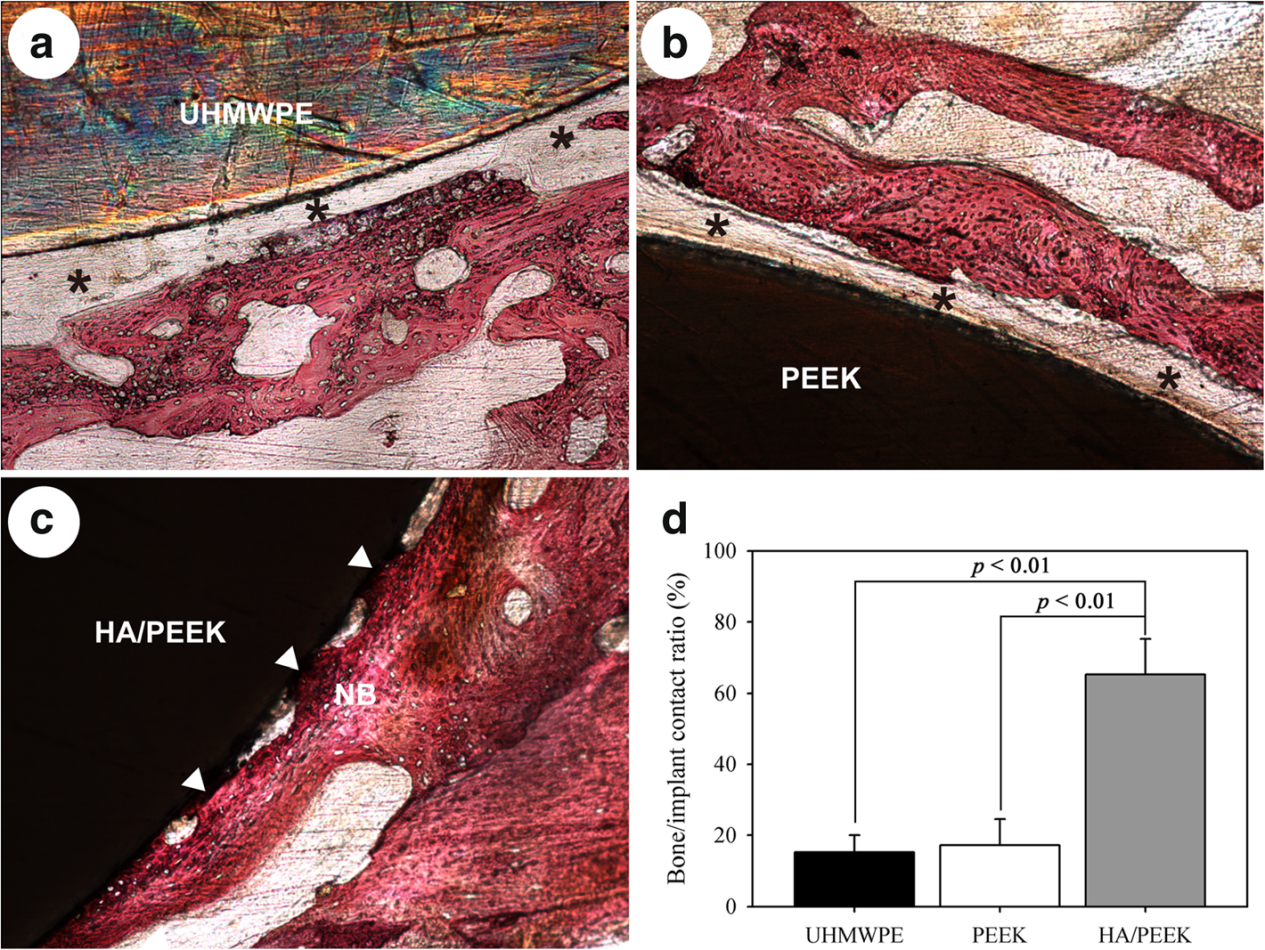
Histological observation after 8 weeks of implantation: **a** UHMWPE, **b** PEEK, **c** HA/PEEK, **d** quantitative analysis of bone/implant contact ratio. The black asterisks indicate the fibrous connective tissue, and the white arrows indicate the bone contact

Figure 11 shows the histological observations after 8 weeks of implantation. A layer of fibrous connective tissue was observed around UHMWPE (Fig. 11a) and PEEK (Fig. 11b), and numerous new bone tissues were in close contact with the surface of the HA/PEEK composite (Fig. 11c). Quantitative analysis showed that the bone/implant contact ratio of the HA/PEEK composite was apparently higher than those of UHMWPE (*p* = 0.001) and PEEK (*p* = 0.002). Therefore, UHMWPE and PEEK were wrapped with fibrous connective tissue after implantation for 8 weeks, but a mass of new bone formed at the bone/implant interface around the HA/PEEK composite.

**Fig. 12:**
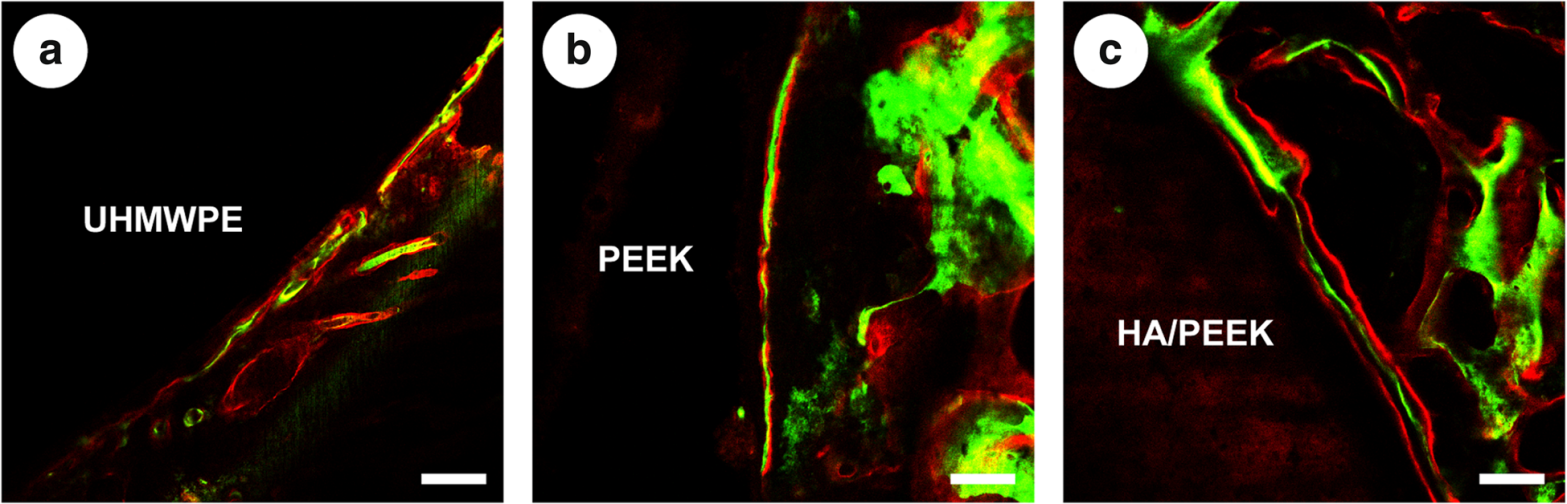
New bone formation labeled by alizarin red (red) and calcein (green): **a** UHMWPE, **b** PEEK, and **c** HA/PEEK. The scale bar represents 100 μm

The new bone formation was labeled with alizarin red and calcein and observed by CLSM. The results of the new bone formation are shown in Fig. 12. Three fluorescent lines overlapped together around the UHMWPE and PEEK, and the green line and the two red lines were separated (Fig. 12) around the HA/PEEK composite. These results demonstrated that more new bone formed around the HA/PEEK composite than around UHMWPE and PEEK.

## Discussion

As a promising thermoplastic material, PEEK possesses good processability, stable chemical properties, and favorable biocompatibility with an elastic modulus that is close to that of human cortical bone [3, 25]. However, PEEK is hydrophobic and bio-inert, which has limited its broad application [4, 5]. Composites that possess tailored biological and mechanical properties can be produced by combining polymers and bioactive inorganic phases [26]. In this study, HA particles were incorporated into the PEEK matrix via a compounding and injection-molding process, and the bioactivity of the prepared HA/PEEK composite was evaluated by in vitro and in vivo studies.

The tensile tests revealed that the elastic moduli of the HA/PEEK composites increased as the HA content increased. This result is primarily due to the modulus of PEEK being 3 GPa with the modulus of HA being as high as 85 GPa [11]. The elastic moduli of the PEEK composites with a HA content of 30% and 40% fell within the lower range of the elastic modulus of cortical bone (7–30 GPa [22]). However, the tensile strength decreased with increasing HA content in the PEEK composites, suggesting loss of ductility. The tensile strength of the 40% HA/PEEK composite was below the lower range of cortical bone (50–150 MPa [22]). This result was due to the inherent characteristics of monolithic HA (i.e., high stiffness and brittleness) [27]. A comparison between composites with 20% and 30% HA indicated that the 30% composite exhibited a higher elastic modulus and slightly lower tensile strength. The values of the elastic modulus and tensile strength of the 30% HA/PEEK composite were higher than the lower range of those for cortical bone. Therefore, 30 wt% was chosen as the optimal HA content. Ma et al. [14, 15] successfully prepared an HA/PEEK composite via an in situ synthetic process, and the composite exhibited an excellent improvement in mechanical properties and bonding between HA and PEEK. The strong bonding between HA particles and PEEK matrix in Ma’s study probably was due to physical factors, such as molecules chain wrapping and the interlock effect of PEEK molecules on HA surface, while the interaction between the HA particle and the PEEK matrix in this study is mechanical interlocking between the two phases [16].

Attachment, proliferation, and differentiation of osteoblasts on the implant surface are necessary for osseointegration between the implant and the bone tissue [28]. To investigate the in vitro bioactivity of the materials, the cell attachment, proliferation, spreading, and ALP activity of MC3T3-E1 cells on the surfaces of the HA/PEEK composite, pure PEEK and UHMWPE were investigated. The HA/PEEK composite promoted cell attachment and proliferation of MC3T3-E1 cells on the material surface. Based on the results from the SEM and CLSM observations, the cells on the HA/PEEK composite exhibited a higher spreading efficiency with more pseudopods that were anchored at the material surface and connected neighboring cells, compared to those on UHMWPE and pure PEEK. In addition to the initial attachment and proliferation, the subsequent ALP activity is also critical factor for bone-implant interfacial osseointegration [29]. In our study, the results revealed that the cells on the HA/PEEK composite exhibited higher ALP activity compared to those on UHMWPE and pure PEEK. Zhang et al. [12] manufactured HA/PEEK composites via the selective laser sintering (SLS) technique, and they found that the SLS-treated HA/PEEK supported osteoblast growth and that composites with higher HA contents exhibited enhanced cell proliferation and osteogenic differentiation (increased ALP activity, and produced more osteocalcin).

PEEK is hydrophobic due to its hydrophobic aromatic ring and polyester functional groups [30], and HA is hydrophilic due to its hydroxyl groups [31]. The HA/PEEK composite was more hydrophilic than UHMWPE and pure PEEK. The hydrophobic surface of pure PEEK hinders cell attachment, leading to its separation from the bone. Several studies have employed a HA coating or HA composite to convert hydrophobic substrate to hydrophilic surface material [17, 32, 33]. In addition, HA possesses the ability to attract osteoblasts [34] and stimulate cell proliferation and osteogenic differentiation of osteoblast cells [35, 36]. Collectively, the HA incorporation in this study modified the surface of PEEK to produce a hydrophilic and bioactive surface, making it a suitable environment for cell attachment, spreading, proliferation, and differentiation.

All bioactive materials can form a bone-like apatite layer on their surfaces in the living body and bond to bone through this apatite layer [7]. The in vitro bioactivity of a material is often evaluated using the SBF immersion test [13, 21]. In the current study, no changes were observed on the surface of pure PEEK but the HA/PEEK composite induced apatite formation after immersion in SBF for 7 days. In addition, the number and size of the apatite mineral islands increased as the immersion time increased. The surface of the HA/PEEK composite was nearly completely covered by the newly formed apatite after 28 days of immersion. In addition, based on the EDS results, the newly formed ball-like particles on the HA/PEEK composite consisted of apatite containing of Ca and P. Yu et al. [13] prepared HA/PEEK composite by mixing, compaction, and pressureless sintering process and evaluated the bioactivities of HA/PEEK composites with 10, 20, 30, and 40 vol% HA by immersing the composite disks in SBF for 4 weeks. They found the growth rate increased with HA volume fraction, suggesting that the bioactivity of the HA/PEEK composite increased with increasing HA content in the composite. Therefore, the HA/PEEK composite was endowed high bioactivity by adding of HA.

One of the key factors identified in the failure of implants is insufficient osseointegration around the biomaterial immediately after implantation [37]. An implant with bioactivity must have the ability to simulate a biological response to achieve osseointegration at the bone/implant interface [38]. After 8 weeks of implantation, abundant new bone was formed and integrated with the implant surface of the HA/PEEK composite. However, the surfaces of UHMWPE and pure PEEK were surrounded by a layer of fibrous connective tissue. To achieve successful osseointegration, the biomaterials should provide efficient and stable interactions with osteoblasts and form an apatite layer on the surface [7, 39]. The prepared HA/PEEK composite promoted cell attachment, spreading, proliferation, and ALP expression and induced apatite formation in vitro. Histological examinations in vivo indicated that the apatite layer was formed on the ceramic surface early in the implantation period and the bone matrix integrated into the apatite [40]. After being implanted, the HA/PEEK composite may induce the formation of an apatite layer, and the attracted osteoblasts attached onto the surface, proliferated, and differentiated to produce collagen and protein, which mineralized to form new bone at the bone/implant interface. However, due to hydrophobic and bio-inert properties, UHMWPE and PEEK did not exhibit favorable osseointegration, and only fibrous connections eventually formed.

At present, PEEK materials are most widely used as a spinal fusion device in spinal surgery. The HA/PEEK biocomposite prepared in this study is a promising material to be applied in the clinic as a material for spinal fusion device and bioactive artificial joint prosthesis. In addition, this study has some limitations. The connection between micro-scale HA and PEEK in the composite is mainly physical bonding, so increase in the binding strength and long-term stability of the interface needs to be appreciated. In addition, more animal model validation works are needed to expand its clinical application value.

## Conclusion

To improve the bioactivity of PEEK, HA particles were incorporated into the PEEK matrix to prepare a HA/PEEK biocomposite using a compounding and injection-molding technique. By evaluating the tensile properties and elastic moduli of the HA/PEEK composites with different HA contents (0–40 wt%), the 30 wt% HA was considered to be an optimal content. In vitro tests demonstrated that the HA/PEEK composite exhibited improved attachment, proliferation, and spreading, and higher ALP activity than those of UHMWPE and pure PEEK. Additionally, the HA/PEEK composite induced apatite formation after immersion in SBF. The in vivo results indicated that the osseointegration efficiency around the HA/PEEK composite was higher than that around UHMWPE and pure PEEK. All these results confirmed that the bioactivity and osteogenesis of PEEK improved substantially after incorporation of HA.

## Abbreviations

ALP: Alkaline phosphatase
CCK-8: Cell counting kit-8
CLSM: Confocal laser scanning microscope
DAPI: 4, 6-Diamidino-2-phenylindole
E: Elastic modulus
EDS: Energy dispersive spectrometry
FBS: Fetal bovine serum
HA: Hydroxyapatite
HDPE: High-density polyethylene
HMDS: Hexamethyldisilazane
ICP-AES: Inductively coupled plasma atomic emission spectroscopy
OD: Optical density
PAEKs: Polyaryletherketones
PBS: Phosphate-buffered saline
PEEK: Polyetheretherketone
SBF: Simulated body fluid
SEM: Scanning electron microscope
TS: Tensile strength
UHMWPE: Ultra-high molecular weight polyethylene
XRD: X-ray diffraction
